# SARS Patients and Need for Treatment

**DOI:** 10.3201/eid1010.040091

**Published:** 2004-10

**Authors:** Johnny W.M. Chan, Samuel Lee

**Affiliations:** * Queen Elizabeth Hospital, Hong Kong Special Administrative Region, People's Republic of China

**Keywords:** SARS, treatment, letter, Hong Kong

**To the Editor:** We read with interest the case report by Wong et al. (*1*). Three similar cases of serologically confirmed severe acute respiratory syndrome (SARS) were treated in our hospital; all of the patients recovered uneventfully without specific treatment. They had either negative results on polymerase chain reaction (PCR) tests for SARS-associated coronavirus (SAR-CoV), or they were admitted when such rapid diagnostic tests were not yet available; hence, SARS-specific treatment was not prescribed.

The first patient was a 35-year-old, previously healthy female tourist from Guangzhou, China, who was admitted to our hospital in late February 2003. She had visited several family members who had atypical pneumonia; some eventually died from the disease. The patient had fever, chills, and dry cough approximately l week after exposure but experienced no mylagia, diarrhea, or shortness of breath. On physical examination, scanty crepitations were heard in her right lower chest, and chest radiographs showed right lower zone consolidation. Blood tests showed a slightly low platelet count of 119 x 10^9^/L and mildly elevated alanine transaminase at 59 U/L (normal <55 U/L), but total and differential leukocyte counts were normal. Tests for etiologic agents included blood and sputum bacterial cultures; sputum for acid-fast bacilli; and nasopharyngeal aspirates for influenza, parainfluenza, adenovirus, and respiratory syncytical virus. Serologic titers for *Mycoplasma*, *Chlamydia psittaci*, and *Legionella* were negative. Reverse transcription (RT)-PCR tests for SARS-CoV were not available at that time.

Oral clarithromycin and intravenous amoxillin-clavulanate (subsequntly switched to levofloxacin) were prescribed. Her high fever (temperature 39.5°C) lasted for 4 days and then gradually subsided; the radiologic abnormality also improved progressively after the first week. Oxygen supplementation of 2 L/min was necessary for the first 2 days. The diagnosis of SARS was made when the patient's convalescent-phase serum sample, collected 33 days after discharge (day 45 of illness), showed an elevated anti-SARS immunoglobulin (Ig) G titer of 1:800 by immunofluorescence.

The second patient was a 34-year-old, previously healthy man; his father had shared a hospital cubicle with a patient who was subsequently diagnosed with SARS. Fever (temperature 39°C), chills, and rigors developed in patient 2 on December 3, 2003, approximately 4 days after his first hospital visit to his father; he had no cough or gastrointestinal symptoms. Chest radiographs showed right lower zone consolidation. Blood tests showed low platelet count of 91 x 10^9^/L, elevated creatinine kinase (370 U/L), and elevated lactate dehydrogenase levels (1,060 U/L). Total and differential leukocyte counts were normal. Tests for etiologic agents of pneumonia had negative results. His fever (the highest temperature was 39.5°C) subsided after day 2 of admission, with a transient spike on day 11 that coincided with a slight increase in right lower zone consolidation. Both abnormalities subsequently resolved promptly, and no oxygen supplement was necessary. RT-PCR test for SARS-CoV was not available in our hospital when he was admitted. However, SARS was diagnosed when his convalescent-phase serum, collected on day 21 of illness, demonstrated a SARS-CoV IgG titer (by immunofluorescence) of 1:3,200, from an initial baseline of <1:25, taken on day 12 of his illness. No treatment was given, since the patient had already fully recovered when the results arrived.

The last patient was a 74-year-old, previously healthy man, who had visited a sick relative; the relative was later diagnosed with SARS. Fever, chills, and cough developed in our patient 4 days later. Chest radiograph showed left lower and middle zone consolidations. Intravenous ceftriaxone and oral clarithromycin were started. Blood tests showed elevated alkaline phosphatase of 226 U/L and alanine transaminase of 126 U/L. His initial leukocyte count was 18.8 x 10^9^/L with neutrophilia (16 x 10^9^/L, 85.2%) and a normal lymphocyte count of 1.2 x 10^9^/L; platelet count was normal. No causative agent was identified, including by RT-PCR test for SARS-CoV. His fever had subsided upon admission, and serial chest radiographs, liver function, and leukocyte counts showed progressive improvement without specific treatment. The diagnosis of SARS was made from two elevated SARS-CoV IgG levels of both 1:3,200 (by immunofluorescence), taken at days 5 and 24 after his admission (days 19 and 38 of illness).

Although SARS was diagnosed in these three patients retrospectively, and they were not treated with antiviral agents, they were managed in isolation wards. Patients reported adhering to droplet precautions after discharge (mainly, wearing surgical face masks when in close contact with others), and none was believed to have transmitted the virus to others.

SARS can be associated with a substantial death rate (*2*). Ribavirin and systemic corticosteroids were used in our hospital during the SARS epidemic. However, the efficacy of this regimen has not been proven, and concerns exist about side effects of both drugs (*3**,**4*). Some retrospective analyses suggested using lopinavir/ritonavir and integrative Chinese and Western medicine were associated with improved outcomes (*5**,**6*). In vitro (*7**,**8*) and animal (*9*) studies have suggested that interferon and monoclonal antibodies might have some effects on the disease. However, data from randomized controlled trials are lacking. All of our patients had been previously healthy, with no coexisting conditions identified as poor prognostic risk factors (*2**,**10*). These three cases, together with the case of Wong et al. (*1*), suggested that at least a subset of SARS adult patients can have a relatively benign clinical course and uneventful recovery, without any specific treatment other than antimicrobial agents.
